# Effect of deferoxamine and ferrostatin-1 on salivary gland dysfunction in ovariectomized rats

**DOI:** 10.18632/aging.204641

**Published:** 2023-04-06

**Authors:** Yong-Il Cheon, Ji Min Kim, Sung-Chan Shin, Hyung-Sik Kim, Jin-Choon Lee, Gi Cheol Park, Eui-Suk Sung, Minhyung Lee, Byung-Joo Lee

**Affiliations:** 1 Department of Otorhinolaryngology-Head and Neck Surgery, College of Medicine, Pusan National University and Medical Research Institute, Pusan National University Hospital, Busan, Korea; 2 Pusan National University Medical Research Institute, Pusan National University School of Medicine, Busan, Korea; 3 Department of Otorhinolaryngology-Head and Neck Surgery, College of Research Institute for Convergence of Biomedical Science and Technology, Pusan National University Yangsan Hospital, Yangsan, Korea; 4 Department of Life Science in Dentistry, School of Dentistry, Pusan National University, Yangsan, Korea; 5 Department of Otolaryngology-Head and Neck Surgery, Samsung Changwon Hospital, Sungkyunkwan University School of Medicine, Changwon, Korea

**Keywords:** menopause, ferroptosis, xerostomia, deferoxamine, ferrostatin-1

## Abstract

The mechanism underlying xerostomia after menopause has not yet been fully elucidated. This study aimed to investigate the mechanism of xerostomia and the effect of the ferroptosis inhibitors deferoxamine (DFO) and ferrostatin-1 (FER) on salivary gland dysfunction in a postmenopausal animal model. Twenty-four female Sprague–Dawley rats were randomly divided into four groups: a SHAM group (*n* = 6, sham-operated rats), an OVX group (*n* = 6, ovariectomized rats), an FER group (*n* = 6, ovariectomized rats injected intraperitoneally with FER), and a DFO group (*n* = 6, ovariectomized rats injected intraperitoneally with DFO). GPX4 activity, iron accumulation, lipid peroxidation, inflammation, fibrosis, and salivary gland function were analyzed. Recovery of GPX4 activity and a decrease in iron accumulation and cytosolic MDA + HAE were observed in the DFO group. In addition, collagen I, collagen III, TGF-β, IL-6, TNF-α, and TGF-β levels were decreased in the DFO group compared to the OVX group. Recovery of GPX4 activity and the morphology of mitochondria, and reduction of cytosolic MDA + HAE were also observed in the FER group. In addition, decreased expression of inflammatory cytokines and fibrosis markers and increased expression of AQP5 were observed in both the DFO and FER groups. Postmenopausal salivary gland dysfunction is associated with ferroptosis, and DFO and FER may reverse the postmenopausal salivary gland dysfunction after menopause. DFO and FER are hence considered promising treatments for postmenopausal xerostomia.

## INTRODUCTION

Aging is characterized by a time-dependent decrease in body function or loss of adaptation [1]. As aging progresses, there is an increase in the incidence of cancer and cardiovascular, lung, kidney, and digestive diseases, as well as the occurrence of physiological changes such as decreased bone density, decreased epithelial barrier function, and skin atrophy [2–6]. Middle-aged women are prone to menopausal syndromes such as hot flashes, sleep disturbances, depressive moods, decreased libido, osteoporosis, and fatigue [7–10]. In addition, dry mouth, along with dry skin and dry eyes, are typical symptoms that appear after menopause [11]. Postmenopausal women with xerostomia are more prone to food dysphagia, a burning sensation in the oral mucosa, loss of appetite, as well as pronunciation and dental problems, and they may be more susceptible to oral infections [12, 13]. Consequently, xerostomia can reduce an individual’s quality of life.

Xerostomia can be defined as a subjective sensation associated with reduction of lubrication and dehydration of the oral mucosa [14]. Xerostomia is known to be common in elderly people, especially women, and its prevalence is thought to range from 5.5% to 46% [15]. Xerostomia is caused by autoimmune diseases such as Sjögren’s syndrome, a history of drug use, head and neck radiation therapy, diabetes, malnutrition, and psychological problems [16]. In women, postmenopausal hormonal changes are also a cause of xerostomia [17]. Although xerostomia occurs frequently after menopause, the mechanism is not well known.

Treatments for xerostomia include medications, topical agents, and oral rinses that may help relieve symptoms, but the effects are temporary [18]. Hormone therapy has been attempted in postmenopausal xerostomia, but its safety remains controversial [19, 20]. Recently, tissue engineering or regenerative medical treatment using stem cells or exosomes for postmenopausal xerostomia has been attempted, but there is still no standard treatment for postmenopausal xerostomia [21–23].

The mechanisms of menopause-induced xerostomia have been studied to involve salivary gland atrophy, oxidative stress, and cell death [24–26]. However, a clear mechanism for this has not yet been identified. The level of serum iron increase markedly after menopause and estrogen deficiency in postmenopausal women increases the expression of genes involved in lipogenesis [27–29]. The increased serum iron and lipogenesis could induce ROS production, and the authors hypothesized that increased serum iron level and ROS might be related to ferroptosis.

Recently, Kwon et al. reported iron accumulation, increased lipid peroxidation, and decreased GPX4 activity in the salivary gland of ovariectomized rats, and reported the relationship between salivary gland dysfunction and ferroptosis [30]. Although the mechanism by which ferroptosis acts on the salivary gland is not clear, it could be a novel treatment strategy for postmenopausal xerostomia if ferroptosis can be inhibited and salivary gland dysfunction can be restored. However, no study has evaluated the effect of anti-ferroptotic agents on menopause-induced xerostomia. This study aims to investigate the mechanism of menopausal xerostomia and the effect of deferoxamine (DFO) and ferrostatin-1 (FER), known as representative drugs of anti-ferroptotic agents, on the salivary gland dysfunction in the post-menopausal animal model.

## RESULTS

### Food intake, body weight, and serum sex-hormone concentration and sex-hormone receptors

**Figure 1:**
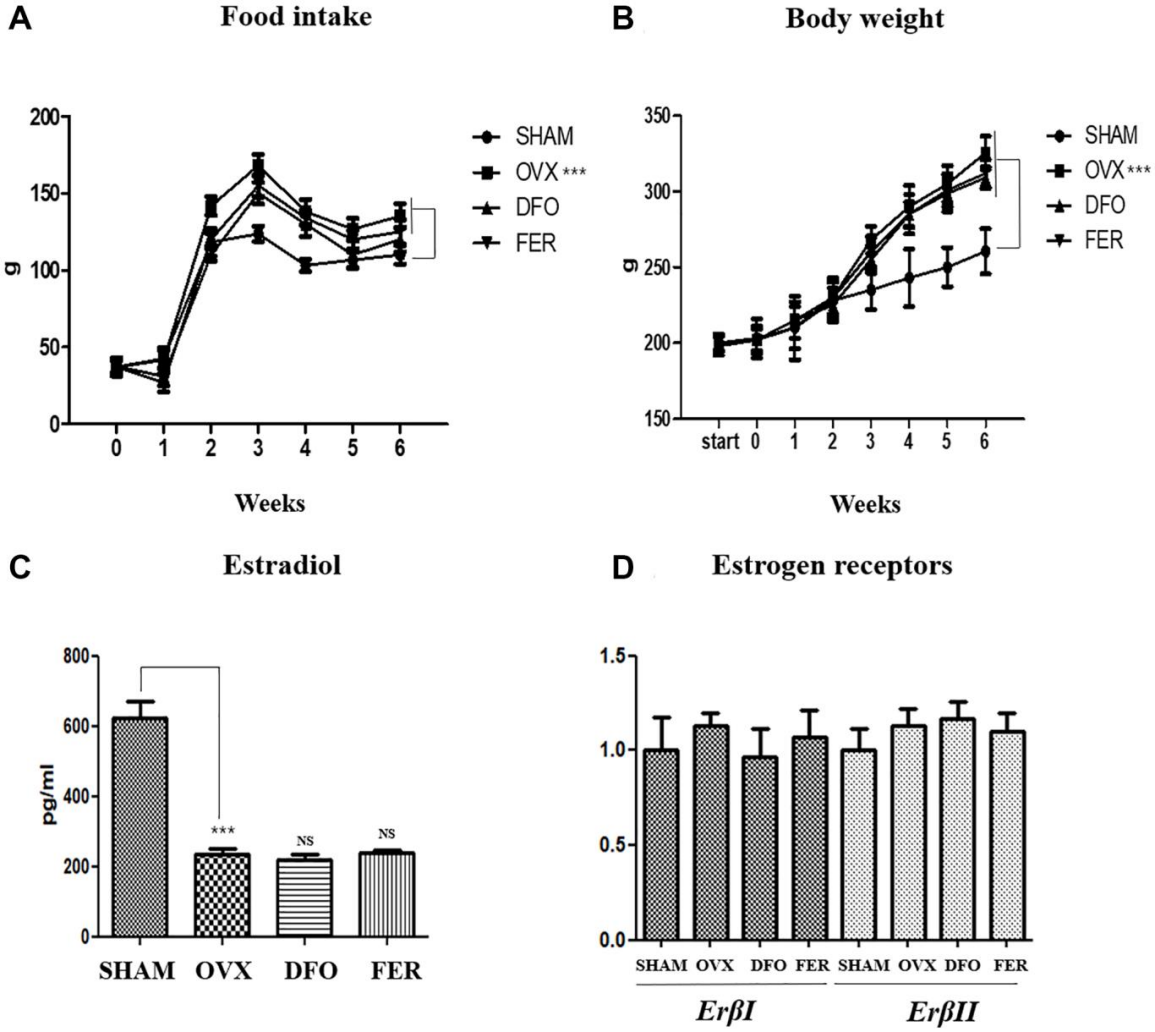
**Food intake, body weight, and sex hormone change.** Food intake (**A**) and body weight (**B**) in each of the four groups. Food intake and body weight increased in the OVX group compared with the SHAM group, but there was no difference in the DFO and FER groups compared with the OVX group. (**C**) The level of serum estradiol concentration in each of the four groups. The serum estradiol levels were lower in the OVX group than in the SHAM group. Neither DFO nor FER treatment affected the serum estradiol level. (**D**) Quantitative real-time PCR analysis of *ErβI* and *ErβII* gene expression in the submandibular gland. The expression of *ErβI* and *ErβII* did not differ between the groups. Two-way ANOVA was performed. ^***^p < 0.001 vs. SHAM group. Abbreviations: NS: not significant; SHAM: sham surgery group; OVX: ovariectomized group; DFO: deferoxamine injection group; FER: ferrostatin-1 injection group; ER: estrogen receptor.

Weight gain is the most common characteristic of menopause. All rat groups were measured weekly for food intake and body weight. The average food intake was higher in the OVX group than in the SHAM group (*p* < 0.001), but there was no difference in the amount of food intake between the DFO and FER groups and the OVX group. The median body weight of the OVX group was significantly higher (*p* < 0.001) than that of the SHAM group. However, no difference was observed between the DFO and the FER group and the OVX group (Figure 1A, 1B and Table 1). The serum estradiol concentration was significantly decreased in the OVX group (*p* < 0.001) compared to that in the SHAM group. However, the estradiol levels in the DFO and FER groups were similar to that observed in the OVX group (Figure 1C). To evaluate the effect of sex hormones on sex hormone receptors, we performed qPCR analysis to determine the expression levels of the estrogen receptor, *Erβ*, in the submandibular gland. The expression levels of *ErβI* and *ErβII* did not differ between any of the groups (Figure 1D). The results indicate that ovariectomy led to a significant reduction in the amount of estradiol, and DFO and FER had no effect on serum estradiol levels. *Erβ* expression was not changed by ovariectomy, and DFO and FER also had no effect on *Erβ* expression in submandibular gland tissue.

**Table 1:** Primers.

**Gene**	**Direction**	**Sequence**
*ErβI*	Forward	GCTTCGTGGAGCTCAGCCTG
	Reverse	AGGATCATGGCCTTGACACAGA
*ErβII*	Forward	GAAGCTGAACCACCCAATGT
	Reverse	CAGTCCCACCATTAGCACCT
*Tnfα*	Forward	GGTCAACCTGCCCAAGTACT
	Reverse	CTCCAAAGTAGACCTGCCCG
*Il-6*	Forward	ATCTGCCCTTCAGGAACAGC
	Reverse	GAAGTAGGGAAGGCAGTGGC
*Col1a1*	Forward	CAGGATGCAGTCCCTGAAAT
	Reverse	GAGGTGGCCTAGGTGGTGTA
*Col3a*	Forward	GGCCCTGTGTGTACTGGTCT
	Reverse	AGCATCAGAGGGAGTGAGGA
*Tgf-βI*	Forward	GACGTTCGCCATAACCAAGT
	Reverse	CTGCAGGTTCTCAATGCAAA
*Tgf-βII*	Forward	CCAATCACGCAATAGTTCTGG
	Reverse	CGCTGTATCGTATGGCGAT
*Aqp3*	Forward	AATTGTCTGGAGCCCACTTG
	Reverse	CAGCTTGATCCAGGGCTCTC
*Aqp5*	Forward	CATGAACCCAGCCCGATCTT
	Reverse	AGAAGACCCAGTGAGAGGGG
*Amy1*	Forward	GCAACCAAGTAGCTTTTGGCA
	Reverse	TGCCATCGACTTTGTCTCCAG
*Gapdh*	Forward	ATCAAGAAGGTGGTGAAGCA
	Reverse	AAGGTGGAAGAATGGGAGTTG

### Lipid deposition

**Figure 2:**
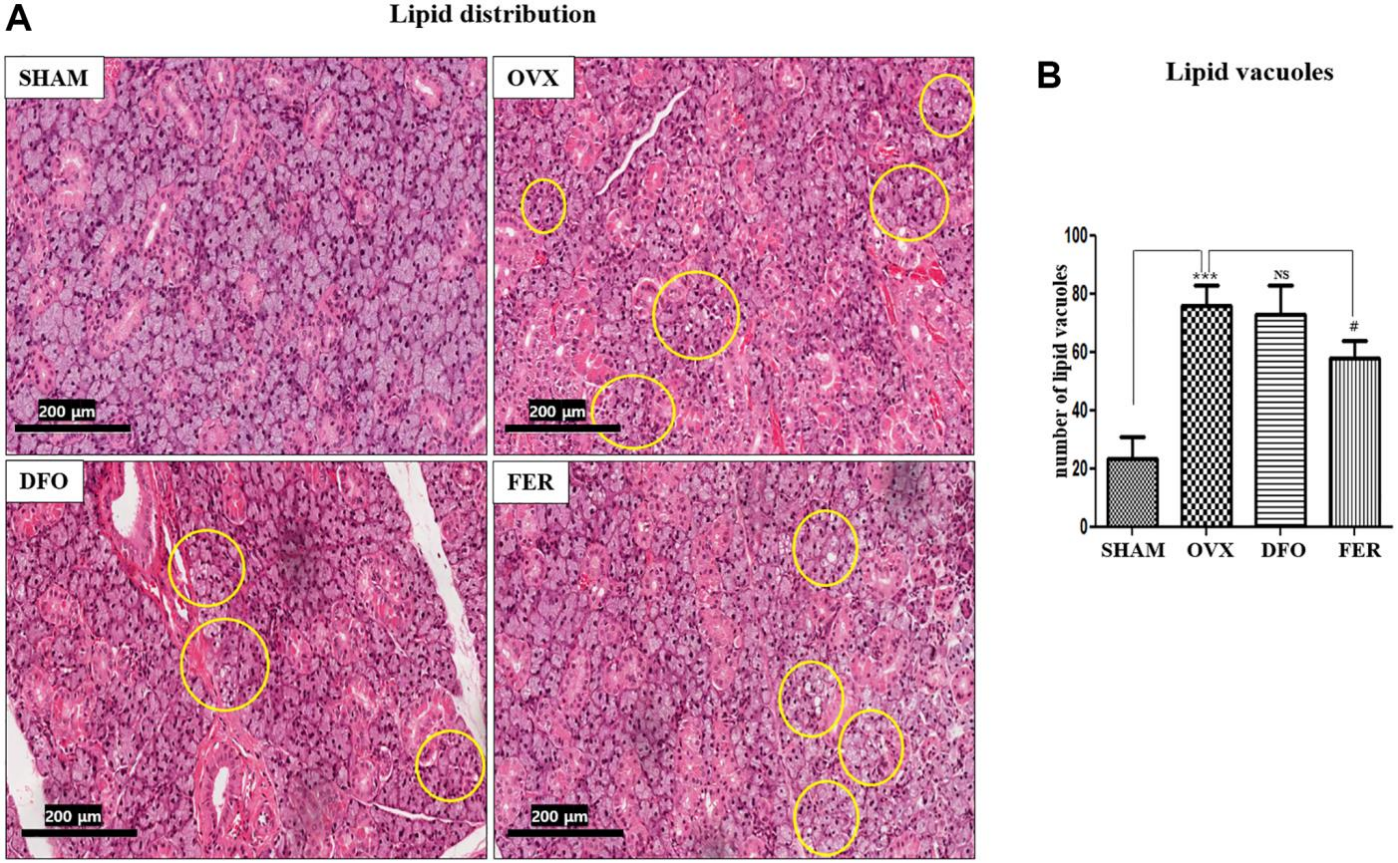
**Lipid deposition in the submandibular gland.** (**A**) Lipid deposition in the submandibular gland (H&E staining, 40×). Yellow circles indicate lipid vacuoles. The lipid distribution increased in the OVX group and decreased in the FER group, but there was no significant difference in the DFO group. (**B**) Morphometric analysis of lipid vacuoles in the SHAM, OVX, DFO, and FER groups. Lipid vacuoles were significantly increased in the OVX group than in the SHAM group. The FER group exhibited decreased lipid vacuoles compared with the OVX group. Two-way ANOVA was performed. ^***^p < 0.001 vs. SHAM group, ^#^*p* < 0.05, vs. OVX group. Abbreviations: NS: not significant; SHAM: sham surgery group; OVX: ovariectomized group; DFO: deferoxamine injection group; FER: ferrostatin-1 injection group.

Lipid deposition was observed in the submandibular glands. The results showed that the lipid distribution was significantly higher in the OVX group than in the SHAM group (*p* < 0.001), and the FER group exhibited a decrease compared to the OVX group (*p* < 0.05) (Figure 2A). The degree of lipid deposition was quantified by counting the number of lipid vacuoles (Figure 2B).

### Inhibitory effect on ferroptosis

**Figure 3:**
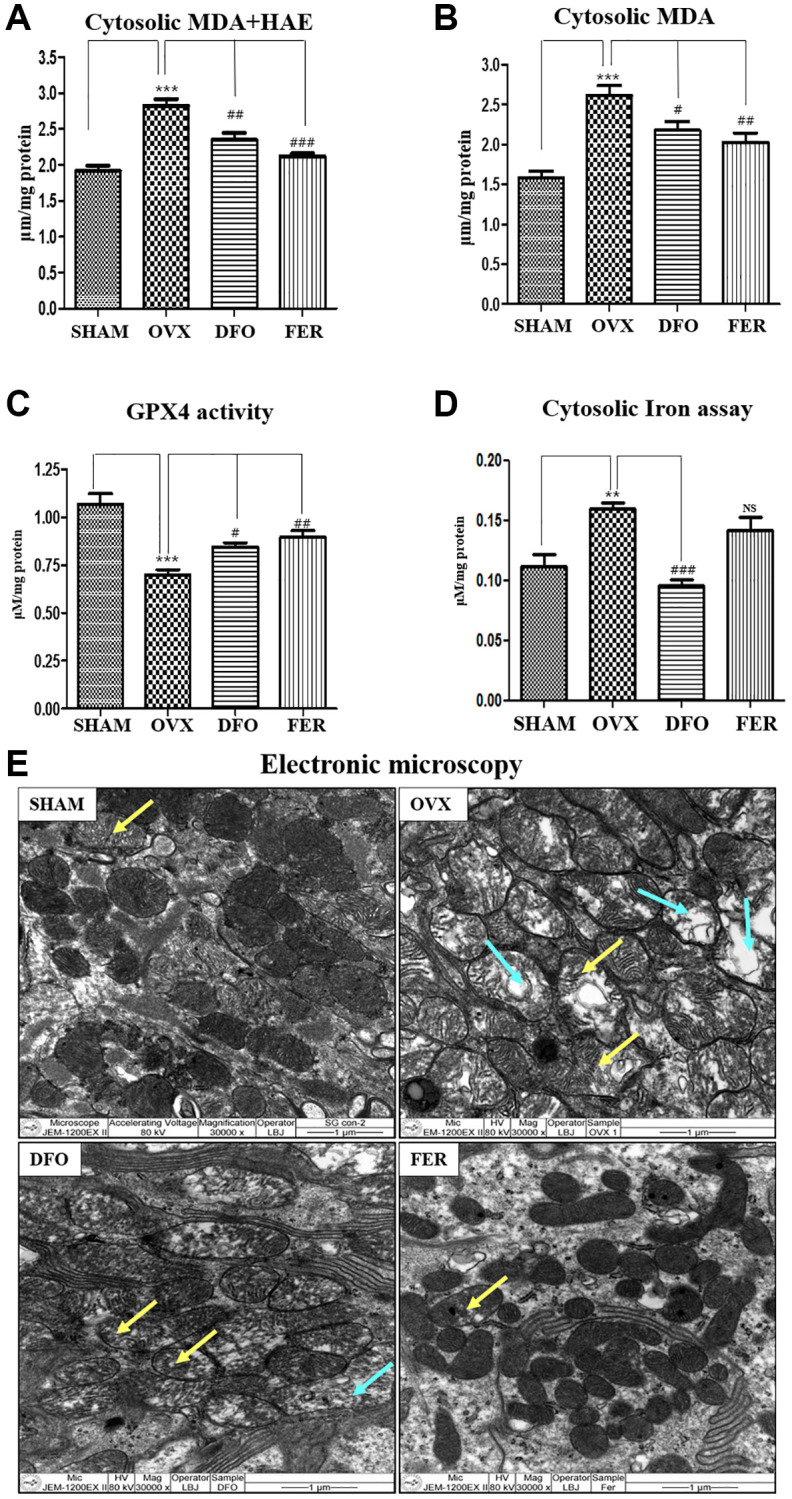
**Inhibitory effect on ferroptosis.** Cytosolic MDA (**A**) and cytosolic MDA + HNE (**B**) concentrations in the submandibular gland tissue. Compared to the SHAM group, the cytosolic MDA and MDA + HAE concentrations in the submandibular gland increased in the OVX group (*p* < 0.001) and decreased in the DFO group (MDA + HAE; *p* < 0.01 and MDA; *p* < 0.05) and FER group (MDA + HAE; *p* < 0.001 and MDA; *p* < 0.01) (Figure 3A and 3B). (**C**) GPX4 activity. GPX4 activity was reduced in the OVX group (*p* < 0.001), while the DFO (*p* < 0.05) and FER (*P* < 0.01) groups exhibited increased GPX4 activity. (**D**) Cytosolic iron content. Compared to the SHAM group, the cytosolic iron level increased in the OVX group and significantly decreased in the DFO group. (**E**) Electron microscopy images of mitochondria in the submandibular gland. The yellow arrow for indicating mitochondrial swelling, and blue arrow for indicating mitochondrial degeneration In the SHAM group, there was clear condensation of mitochondria and the shape was normal, but in the OVX group, the shape was irregular and the integrity was decreased. In the DFO and FER groups, mitochondrial recovery could be confirmed morphologically. Two-way ANOVA was performed. ^**^p < 0.01, ^***^p < 0.001 vs. SHAM group, ^#^*p* < 0.05, ^##^*p* < 0.01, ^###^*p* < 0.001 vs. OVX group. Abbreviations: NS: not significant; SHAM: sham surgery group; OVX: ovariectomized group; DFO: deferoxamine injection group; FER: ferrostatin-1 injection group; MDA: malondialdehyde; HAE: 4-hydroxynonenal; GPX4: glutathione peroxidase 4.

MDA and HAE are lipid peroxidation products and common ferroptosis markers. Compared with the SHAM group, the cytosolic MDA and MDA + HAE concentrations of the submandibular gland increased in the OVX group and decreased in the DFO and FER groups (*p* < 0.05) (Figure 3A and 3B). GPX4 (glutathione peroxidase 4) activity is important for regulation of the ferroptosis pathway. GPX4 activity was significantly reduced in the OVX group and recovered in the DFO and FER groups (Figure 3C). To assess the iron content, which is a key marker of ferroptosis, we assayed the iron level in the submandibular gland. Compared to the SHAM group, the cytosolic iron level increased in the OVX group (*p* < 0.01) and was significantly decreased in the DFO group (*p* < 0.001). There was no difference between the FER group and the OVX group (Figure 3D). We observed the status of mitochondria in the submandibular gland because mitochondria play an important role in oxidative metabolism. In the SHAM group, there was clear condensation of mitochondria and their shape was normal, but in the OVX group, the shape was irregular and the integrity was decreased. In the DFO and FER groups, mitochondrial recovery was confirmed morphologically (Figure 3E).

### Inflammatory cytokines

**Figure 4:**
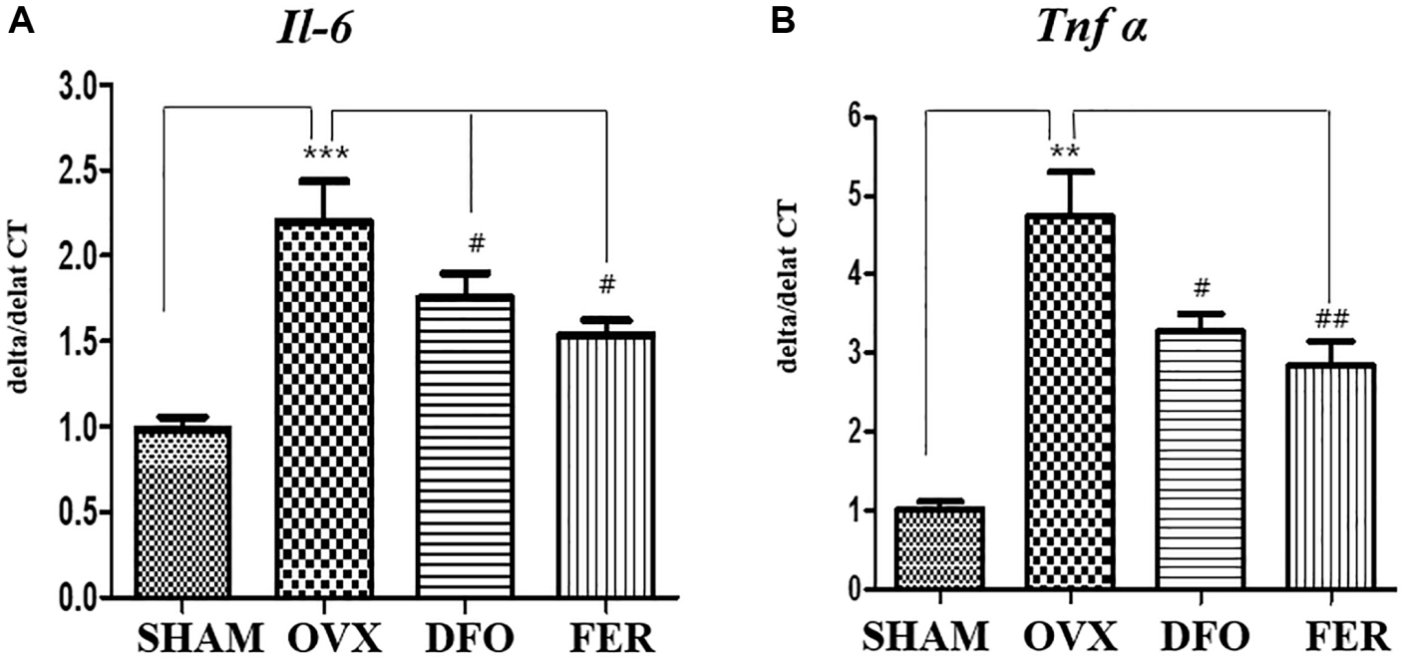
**Inflammatory cytokine secretion in the submandibular gland.** Quantitative real-time PCR analysis of *Il-6* (**A**) and *Tnf-α* (**B**) gene expression in the submandibular gland. The expression levels of *Il-6* and *Tnf-α* mRNA were increased in the OVX group and decreased in the DFO and FER groups. Two-way ANOVA was performed. ^**^p < 0.01, ^***^p < 0.001 vs. SHAM group, ^#^*p* < 0.05, ^##^*p* < 0.01 vs. OVX group. Abbreviations: NS: not significant; SHAM: sham surgery group; OVX: ovariectomized group; DFO: deferoxamine injection group; FER: ferrostatin-1 injection group; Il-6: interleukin 6; Tnf-α: tumor necrosis factor-alpha.

The inhibitory effects of DFO and FER on the secretion of pro-inflammatory cytokines were evaluated. As shown in Figure 4A, the expression level of interleukin 6 (*Il-6*) mRNA was increased in the OVX group (*p* < 0.001) and decreased in the DFO (*p* < 0.05) and FER groups (*p* < 0.05). In addition, the level of tumor necrosis factor-alpha (*Tnf-α*) mRNA was also increased in the OVX group (*p* < 0.01) and decreased in the DFO (*p* < 0.05) and FER (*p* < 0.01) groups (Figure 4B). These results indicate that DFO and FER can suppress the increased secretion of pro-inflammatory cytokines by OVX.

### Fibrosis markers

**Figure 5:**
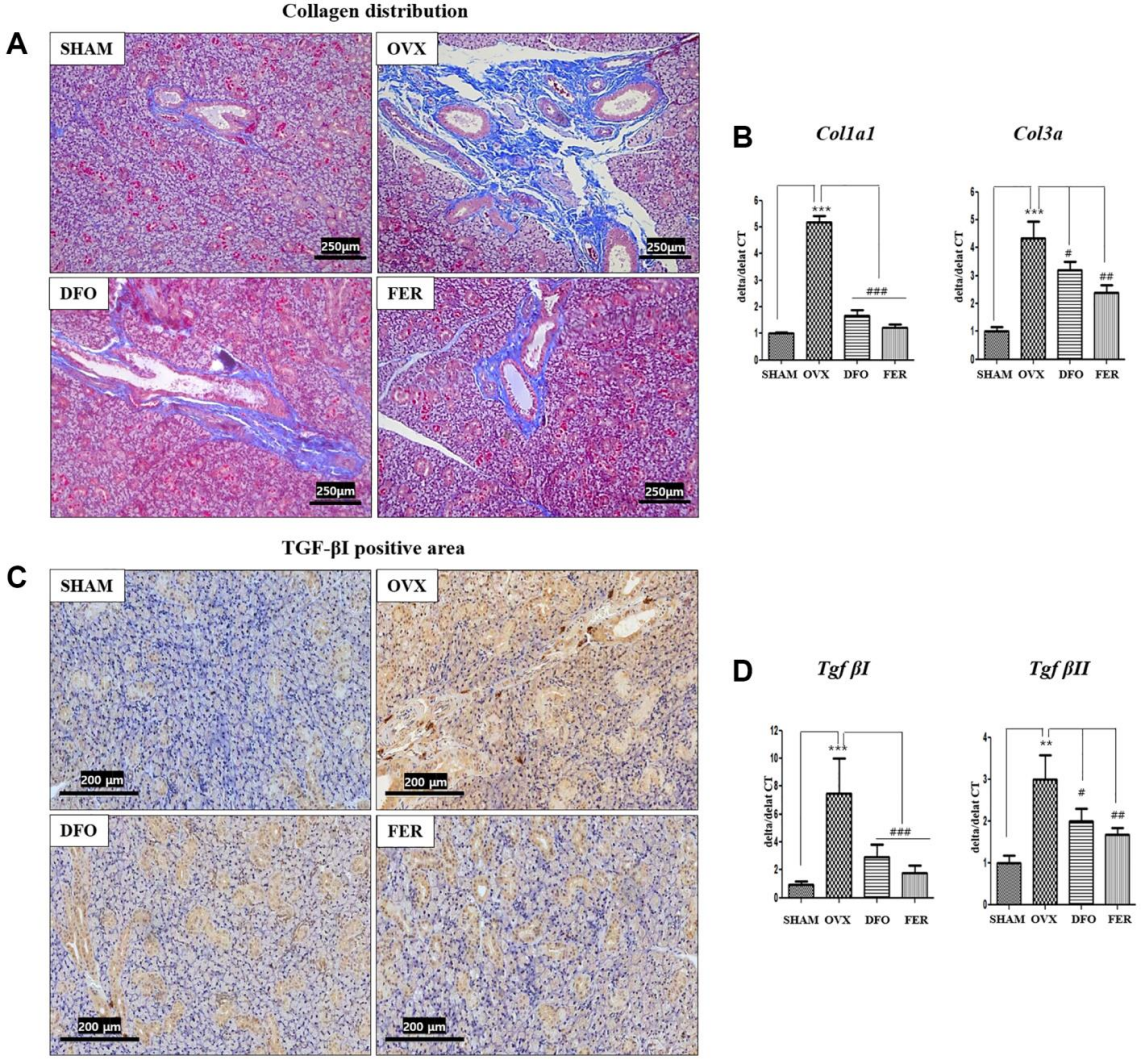
**Fibrosis in the submandibular gland.** (**A**) Collagen distribution in the salivary gland tissue. The fibrotic area was significantly larger in the OVX group than in the SHAM group, while in both the DFO and the FER group it was smaller than in the OVX group. (**B**) Expression of *Col1a1* and *Col3a* mRNAs. The expression of collagen type I alpha 1 chain (*Col1a1*) and collagen type III alpha chain (*Col3a*) mRNAs also increased in the OVX group (*p* < 0.001) and decreased in the DFO (*Col1a1*; *p* < 0.001 and *Col3a*; *p* < 0.05) and FER (*Col1a1*; *p* < 0.001 and *Col3a*; *p* < 0.01) groups. (**C**) Immunohistochemical assessment of TGF-βI levels. The brown staining reflects TGF-βI expression. The TGF-βI staining was higher in the OVX group than in the SHAM group and decreased in both the DFO and the FER group compared with that in the OVX group. (**D**) Expression of *Tgf-βI* and *Tgf-βII* mRNAs. The expression of *Tgf-βI* and *Tgf-βII* mRNAs increased in the OVX group (*Tgf-βI*; *p* < 0.001 and *Tgf-βII*; *p* < 0.01) and decreased in the DFO (*Tgf-βI*; *p* < 0.001 and *Tgf-βII*; *p* < 0.05) and FER (*Tgf-βI*; *p* < 0.001 and *Tgf-βII*; *p* < 0.01) groups. Two-way ANOVA was performed. ^**^p < 0.01, ^***^p < 0.001 vs. SHAM group, ^#^*p* < 0.05, ^##^*p* < 0.01, ^###^*p* < 0.001 vs. OVX group. Abbreviations: SHAM: sham surgery group; OVX: ovariectomized group; DFO: deferoxamine injection group; FER: ferrostatin-1 injection group; Col1a1: Collagen type I alpha 1 chain; Col3a: Collagen type III alpha chain; TGF: Tumor growth factor.

Masson’s trichrome staining showed that the degree of fibrosis in the submandibular gland tissue was significantly higher in the OVX group than that in the SHAM group. However, both the DFO and FER groups exhibited significantly decreased fibrosis (Figure 5A). The expression of collagen type I alpha 1 chain (*Col1a1*) and collagen type III alpha chain (*Col3a*) mRNAs was also significantly increased in the OVX group and decreased in the DFO and FER groups (Figure 5B). Immunohistochemical analysis showed that the level of TGF-βI was higher in the OVX group and lower in the DFO and FER groups (Figure 5C). The expression *of Tgf-βI* and *Tgf-βII* mRNAs also significantly increased in the OVX group and decreased in the DFO and FER groups (Figure 5D).

### Expression of aquaporin and amylase

**Figure 6:**
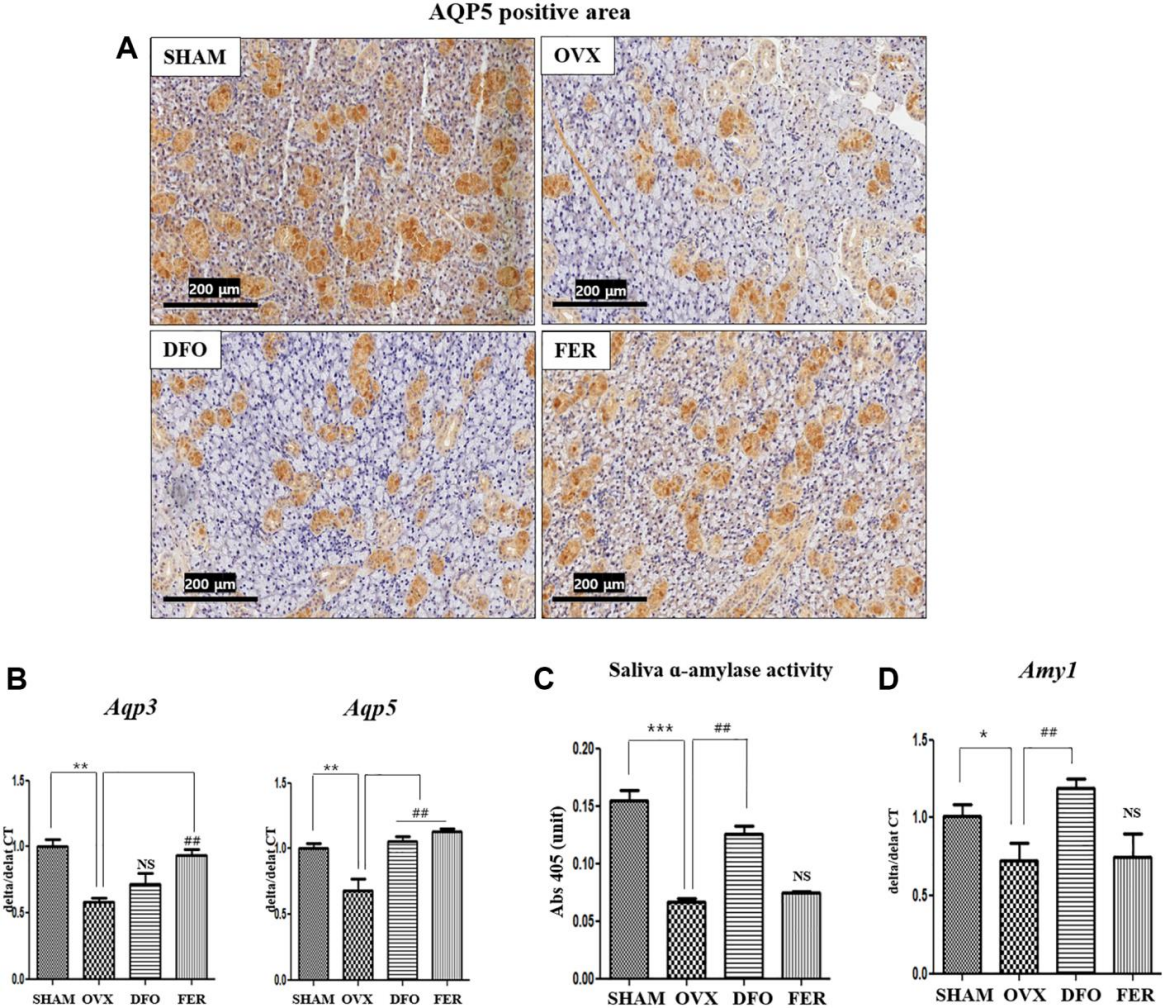
**Aquaporin and amylase in the submandibular gland.** (**A**) Immunohistochemical assessment of AQP5 expression. The brown staining reflects AQP5 expression. The expression AQP5 was lower in the OVX group than in the SHAM group and increased in both the DFO and FER groups compared with that in the OVX group. (**B**) Expression of *Aqp3* and *Aqp5* mRNAs. The expression of *Aqp3* mRNA was increased in the FER group compared to the OVX group (*p* < 0.01), but there was no difference in the DFO group, and the expression of *Aqp5* mRNA was increased in both the DFO and FER groups compared to the OVX group (*p* < 0.01). (**C**) Salivary α-amylase activity and (**D**) expression of *Amy1* mRNA. Salivary α-amylase activity and *Amy1* mRNA expression were also significantly increased in the DFO group compared to the OVX group, but there was no difference in the FER group. Two-way ANOVA was performed. ^*^p < 0.05, ^**^p < 0.01, ^***^p < 0.001 vs. SHAM group, ^##^*p* < 0.01 vs. OVX group. Abbreviations: NS: not significant; SHAM: sham surgery group; OVX: ovariectomized group; DFO: deferoxamine injection group; FER: ferrostatin-1 injection group.

To determine whether ferroptosis inhibitors could improve submandibular gland function, we assayed aquaporin (AQP) and α-amylase levels, as they are important for saliva generation. Immunohistochemical analysis showed that the level of AQP5 decreased in the OVX group and significantly increased in the DFO and FER groups (Figure 6A). Quantitatively, the expression of *Aqp3* mRNA was increased in the FER group compared to the OVX group (*p* < 0.01), but there was no difference with the DFO group. The expression of *Aqp5* mRNA was increased in both the DFO and FER (*p* < 0.01) groups compared to the OVX group (*p* < 0.01) (Figure 6B). Salivary α-amylase activity decreased in the OVX group (*p* < 0.001) and increased in the DFO group (*p* < 0.01) (Figure 6C). Quantitatively, the expression of alpha-amylase 1 (*Amy1α*) mRNA was decreased in the OVX group (*p* < 0.05) and increased in the DFO group (*p* < 0.01) (Figure 6D). These results suggest that the ferroptosis inhibitors can reverse the submandibular gland dysfunction that occurs after menopause.

## DISCUSSION

Menopause is a normal aging process and is accompanied by changes in the body due to hormonal fluctuations. Decreased female hormone levels can result in changes in lipid metabolism, increased iron deposition, abdominal obesity, cardiovascular disease, hot flashes, osteoporosis, and other menopausal syndromes [27, 31]. In addition, dry skin, dry eyes, and xerostomia can occur [32]. Although xerostomia significantly affects the quality of life of postmenopausal women, the exact mechanism by which this occurs has not yet been reported. Recently, it was reported that ferroptosis in the salivary gland may be related to the xerostomia that occurs after menopause [30]. However, no studies to date have used anti-ferroptosis drugs to investigate the mechanisms underlying postmenopausal salivary gland dysfunction.

Ferroptosis was identified in 2012 as a type of cell death [33], and it is defined as a process of programmed cell death characterized by increased iron accumulation, lipid peroxidation, and decreased GPX4 activity. Morphologically, condensation of nuclei does not occur in ferroptosis, but mitochondrial contraction and mitochondrial outer membrane rupture do occur [34]. Several studies have reported the relationship between ferroptosis and various diseases, including neurodegenerative diseases, stroke, sepsis, and kidney and liver diseases [35, 36]. In this study, we confirmed that salivary gland function was reduced after OVX and improved after the administration of ferroptosis inhibitors (DFO and FER) in a postmenopausal animal model.

Ferroptosis inhibitors can act at multiple stages of the ferroptosis pathway, inhibiting several factors including iron accumulation, GPX4 activity, and lipid peroxidation. Entities that block ferroptosis can be classified into class I and class II inhibitors [37, 38]. Class I substances inhibit the accumulation of iron, and they include compounds such as deferoxamine (DFO), mesylate, and 2,2′-pyridine. Class II inhibitors, such as ferrostatin-1 (FER), liprostatin-1, SRS16-86, and vitamin E play a role in inhibiting lipid peroxidation. In this study, among various ferroptosis inhibitors, DFO is a representative of class I, and Ferrostatin-1 is one of the most representative agents of class II. So, by selecting the most representative drug in each class, we investigated the effects of ferroptosis inhibitors on salivary gland dysfunction in ovariectomized rat model.

DFO is an iron chelator approved by the FDA in 1968 and has a hexadentate structure that exhibits a high affinity for iron. When DFO comes into contact with iron, its structure is altered, and it binds to iron ions via three hydroxamic acid groups, thereby decreasing the amount of iron accumulated in the body [39]. In this study, administration of DFO resulted in recovery of GPX4 activity and reduction of iron accumulation and lipid peroxidation. These results are similar to those of other studies that reported an increase in GPX4 activity, a decrease in iron, and a decrease in ROS by DFO administration [40–42]. In addition to the suppression of ferroptosis by DFO administration, decreased expression of inflammatory cytokines and fibrosis markers and increased expression of AQP5 and salivary α-amylase activity were observed. These results suggest that DFO is a useful drug that can be applied to the treatment of postmenopausal xerostomia by inhibition of ferroptosis. However, administration of DFO is known to cause side effects such as visual and auditory disorders, growth retardation, allergic reactions, and pulmonary and neurological disorders [43–47]. Therefore, long-term administration of DFO is controversial and requires regular monitoring of ferritin levels and side effects.

FER is a synthetic arylalkylamine compound that is a potent inhibitor of ferroptosis [47]. FER plays a role in ferroptosis through inhibition of lipid peroxidation by trapping transport chain radicals [48]. In this study, the recovery of GPX4 activity and the status of mitochondria, as well as reduction of lipid peroxidation were observed when FER was administered in a postmenopausal animal model. These results are similar to those of other studies reporting an increase in GPX4 activity and a decrease in mitochondrial damage by FER administration [49, 50]. Moreover, similar to the effect of DFO administration, decreased expression of inflammatory cytokines and fibrosis markers and increased expression of AQP3 and AQP5 were observed following FER administration. These results suggest that FER may be involved in restoration of salivary gland function by inhibition of ferroptosis in a postmenopausal animal model.

**Figure 7:**
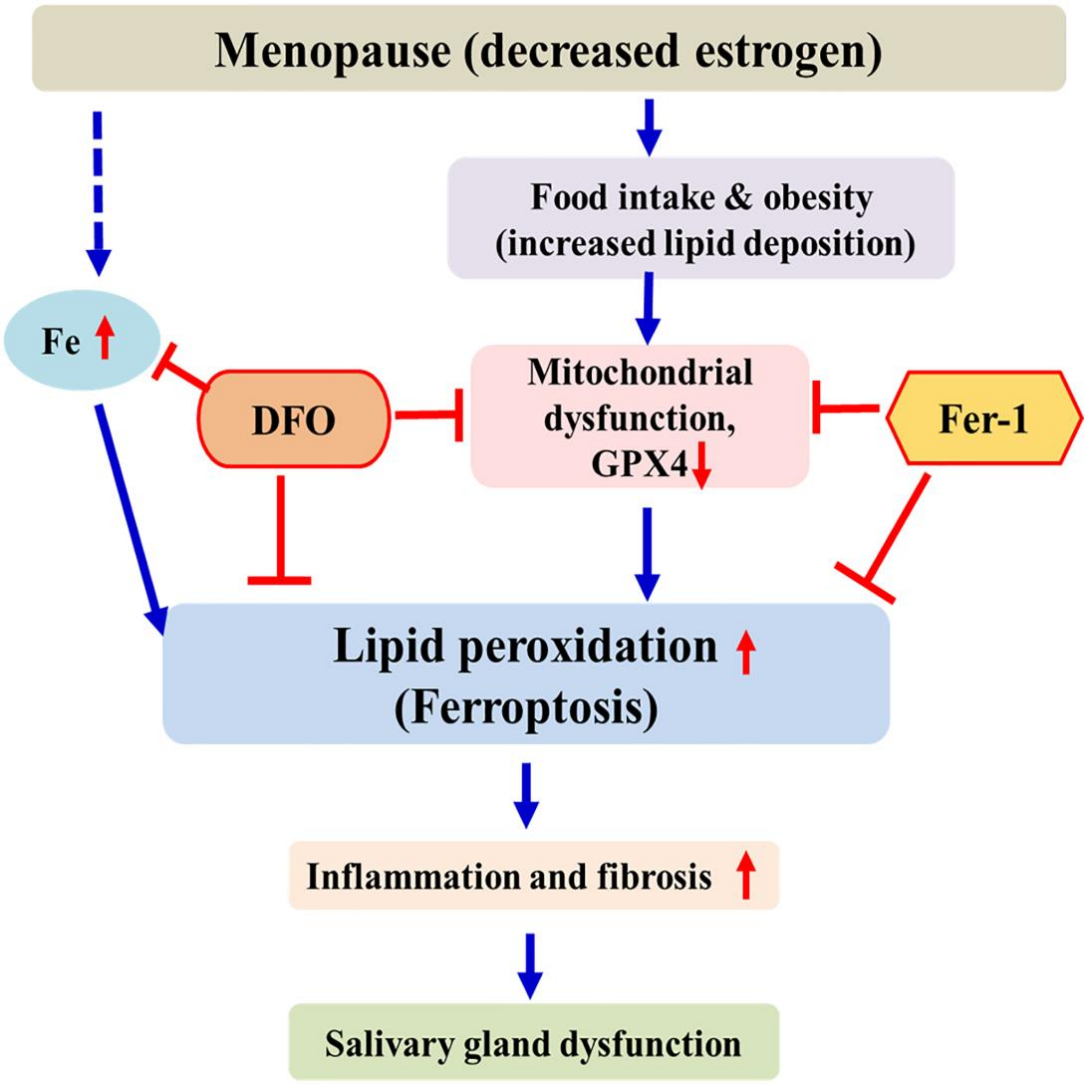
**Schematic diagram of postmenopausal salivary gland dysfunction in ovariectomized rats.** After menopause, iron and lipids accumulate in the salivary glands and GPX4 activity decreases. This promotes lipid peroxidation and induces ferroptosis. DFO and FER inhibit the ferroptosis pathway, thus reducing salivary gland dysfunction.

This study can be summarized as follows. As menopause progresses, accumulation of iron and lipids occurs in the salivary glands. This can promote lipid peroxidation, which leads to ferroptosis. Ferroptosis induces tissue inflammation and fibrosis of the salivary gland, leading to decreased salivary gland function. These results are similar to those reported by Kwon et al. [30]. As a chelating agent, DFO interferes with the ferroptosis pathway by inhibiting iron accumulation, thereby increasing GPX4 activity and decreasing lipid peroxidation. FER exerts anti-ferroptosis effects by increasing GPX4 activity and by inhibiting lipid peroxidation. (Figure 7). This is the first study to investigate the effect of ferroptosis inhibitors on the salivary glands of ovariectomized rats. Therefore, ferroptosis is involved in salivary gland dysfunction after menopause, and ferroptosis inhibitors may present a new treatment method for postmenopausal xerostomia. Our results provide a background for applying various antioxidant agents other than DFO and FER to the treatment of postmenopausal salivary gland dysfunction. Further studies are needed for clinical application of these findings.

A number of studies have shown that various antioxidants, such as dietary nitrate, *Cimicifuga racemosa* methanol extracts, oligonol and echinochrome A help restore salivary gland function after menopause [25, 51, 52]. These substances are thought to reverse salivary gland dysfunction after menopause by inhibiting apoptosis, increasing Cu-Zn SOD activity, and reducing mitochondrial biogenesis or lipid peroxidation. However, there have been no studies on these antioxidants and ferroptosis in menopause xerostomia. Future studies on the association of antioxidants with ferroptosis are hence needed.

However, this study also has limitations. First, since an *in vitro* model that can mimic menopause is not possible, there is a limit to accurate elucidation of the mechanism of the effect of DFO and FER. Second, in order to use DFO and FER in actual clinical practice, additional studies on safety and optimal dosing are needed. In the absence of a standard treatment for postmenopausal dry mouth, this study is expected to be helpful in understanding the mechanism of postmenopausal salivary gland dysfunction and developing a treatment for postmenopausal dry mouth.

## CONCLUSION

Postmenopausal salivary gland dysfunction is associated with ferroptosis. This is the first study to investigate the effect of ferroptosis inhibitors (DFO and FER) on the salivary glands of ovariectomized rats. DFO and FER are considered promising treatments for postmenopausal xerostomia.

## MATERIALS AND METHODS

### Establishment of the animal model

Twenty-four female Sprague-Dawley rats were used in this study (Central Lab, Animal Inc., Seoul, Korea). In order to induce menopause, nine-weeks-old rats that completed sexual maturity were purchased and had a week of acclimatization. Each group was weight-matched at the beginning of the study. The ferroptosis inhibitors deferoxamine and ferrostatin-1 were purchased from Sigma-Aldrich (St. Louis, MO, USA). The rats were randomly divided into four groups. Group I (*n* = 6, sham-operated rats, called SHAM), group II (*n* = 6, ovariectomized rats, OVX), group III (*n* = 6, ovariectomized rats injected with deferoxamine, DFO), and group IV (*n* = 6, ovariectomized rats injected with ferrostatin-1, FER). For ovariectomy surgery, the rats were anesthetized using isoflurane inhalation (3% dissolved in oxygen), and an incision was made at the midline of the abdomen resulting in the bilateral ovaries being revealed. In the OVX group, the ovaries were ligated and cut off bilaterally followed by closure of the abdominal cavity. In the SHAM group, ovariectomy surgery was performed by exposing the ovaries but without excision of the ovaries. Ovariectomized rats were injected intraperitoneally with 100 mg/kg of deferoxamine (DFO) or 2.5 μM/kg of ferrostatin-1 (FER) three times a week for 6 weeks. The study was approved by the Institutional Animal Care and Ethics Committee of Pusan National University Hospital (No. PNUH-2021-179).

### Plasma estradiol concentration

Serum estradiol concentrations were measured using rat-specific estradiol enzyme-linked immunosorbent (ELISA) assay plates coated with a biotin-conjugated binding protein kit purchased from Calbiotech Inc. (Spring Valley, CA, USA). A cardiac puncture was performed, and the blood was centrifuged at 3000 rpm for 30 min. The plasma was separated from the blood collected during exsanguination, immediately frozen in liquid nitrogen, and then stored at −80°C.

### Quantitative PCR

Tissue RNA was extracted using TRIzol^®^ reagent (Life Technologies Inc., Rockville, MD, USA). A reverse transcription kit (Applied Biosystems, Foster City, CA, USA) was used to perform reverse transcription according to the manufacturer’s protocol. Quantitative PCR was performed according to the SYBR^®^ Green PCR protocol (Applied Biosystems). The reaction conditions were: 10 min at 95°C (one cycle); 10 s at 95°C; and 30 s at 60°C (40 cycles). Gene-specific PCR products were continuously measured by an ABI PRISM™ 7900 HT Sequence Detection System (PE Applied Biosystems, Waltham, CT, USA). The primer sequences are presented in Table 2. Normalization consisted of using the differences between the cycle thresholds (delta CT) and the expression level for *Gapdh* to calculate the delta CT/target gene delta CT ratio.

**Table 2:** Food intake and body weight.

	**SHAM**		**OVX**		**DFO**		**FER**	
	**0 weeks**	**6 weeks**	**0 weeks**	**6 weeks**	**0 weeks**	**6 weeks**	**0 weeks**	**6 weeks**
Body weight (g)	202.8 ± 13.0	259.8 ± 14.6	201.8 ± 7.9	325.5 ± 10.9^***^	202.5 ± 7.4	309.4 ± 12.0	201.8 ± 9.5	311.8 ± 9.9
Food intake (g)	37.0 ± 5.9	109.8 ± 6.3	36.0 ± 3.8	134.8 ± 7.8^***^	38.0 ± 3.6	120.0 ± 9.7	36.8 ± 3.7	124.7 ± 8.3

### Staining and immunohistochemistry analysis

The submandibular gland was isolated from each rat, fixed overnight in 4% formalin, and then embedded in paraffin. Cross-sections were prepared for hematoxylin and eosin and Masson’s trichrome staining and immunohistochemistry. For quantitative analyses of TGF-βI and AQP5 expression, deparaffinized sections were incubated for 24 h at 4°C with the following primary antibodies: anti-TGF-βI and AQP5 (200 μg/mL) (Santa Cruz Biotechnology, Dallas, TX, USA). After removal of the primary antibody and rinsing, the sections were incubated with goat anti-rabbit secondary antibody (1:1000) (ENZO Biochem Inc., NY, USA) for 1 h at room temperature) and double-stained with DAB (3,3-diaminobenzidine). Incubation with phosphate-buffered saline supplemented with 1% bovine serum albumin instead of the primary antibody served as a negative control.

### Tissue preparations

Frozen submandibular gland tissues were homogenized in hypotonic lysis buffer using a tissue homogenizer for 20 sec. Homogenates were kept on ice for 15 min, 125 μL of 10% Nonidet P-40 (NP-40) solution was added and mixed for 15 seconds, and the mixture was centrifuged at 14,000 × *g* for 2 min. The supernatants were used as the cytosol fraction. The protein concentration was measured by the bicinchoninic acid (BCA) assay (Life Technologies).

### Lipid peroxidation

Malondialdehyde (MDA)/4-hydroxyalkenals (HAE) concentrations were determined using a Bioxytech LPO-586 Assay Kit (OXIS Health Products, Foster, CA, USA). The kit uses a chromogenic reagent that reacts with the MDA and HAE lipid peroxidation products, yielding a stable chromophore with a maximum absorbance at 586 nm.

### Electron microscopy

The material was pre-fixed with 2.5% glutaraldehyde (4°C, phosphate buffer, pH 7.4) and then post-fixed with 1% osmium tetroxide in the same buffer. The material was dehydrated with a series of graded ethyl alcohols and then embedded in epoxy resin (Epon 812 mixture). Thick sections (1 μm) were stained with 1% toluidine blue for imaging by light microscopy. Thin sections (50~60 nm) were prepared by using an ultramicrotome (EM UC7, Leica) and were double stained with uranyl acetate and lead citrate. The thin sections were examined with a transmission electron microscope (JEM-1200EXII, JEOL). The morphological features of mitochondria were observed by electron microscopy.

### Glutathione peroxidase 4 (GPX4) activity

GPX4 activity was assayed with a Glutathione Peroxidase Assay Kit (Abcam, Cambridge, UK). The assays were performed according to the manufacturer`s instructions. The protein content of the samples was then measured relative to bovine serum albumin standards (Sigma-Aldrich) using the BCA assay.

### Cytosolic iron assay

The concentration of iron in serum was determined with a rat-specific colorimetric iron assay kit from Biovision Incorporation (Spring Valley, CA, USA). Ferric carrier protein dissociates ferric iron into solution in the presence of an acidic buffer. A specific chelate chemical is included in the buffer to block copper ion (Cu^2ᵻ^) interference. After reduction to the ferrous form (Fe^2+^), the iron reacts with Ferene-S to produce a stable-colored complex with a peak absorbance at 593 nm.

### Salivary α-amylase activity

Rats were anesthetized, cotton balls were placed in their mouths, and 2 mg/kg body weight of pilocarpine (Merck, Kenilworth NJ, USA) was injected intraperitoneally to induce saliva secretion. After injection, the mouth was wiped with a cotton swab and filled with cotton balls. In order to prevent saliva loss, a 50 mL tube was placed over the head of the rat and fixed in the anesthesia box, and the head of the rat was installed so that it descended at an oblique angle. In order to avoid errors, this treatment was performed equally for all mice within 5 min, and saliva was collected up to 30 min after pilocarpine injection in the same anesthesia box. After 30 min, the total weight of secreted saliva and (including the weight of cotton balls before and after collection) was evaluated. The α-amylase activity was assayed with an α-amylase activity kit (Sigma-Aldrich).

### Statistical analysis

Unless otherwise stated, all quantitative data are reported as the mean standard errors of the mean from at least three parallel repeats. Two-way analysis of variance (ANOVA) was used to determine significant differences between groups in which *P* < 0.05 was considered statistically significant.
